# Acute vascular effects of carbonated warm water lower leg immersion in healthy young adults

**DOI:** 10.14814/phy2.13046

**Published:** 2016-12-06

**Authors:** Shigehiko Ogoh, Ryohei Nagaoka, Takamasa Mizuno, Shohei Kimura, Yasuhiro Shidahara, Tomomi Ishii, Michinari Kudoh, Erika Iwamoto

**Affiliations:** ^1^ Department of Biomedical EngineeringToyo UniversityKawagoe‐ShiSaitamaJapan; ^2^ School of Health SciencesSapporo Medical UniversitySapporoHokkaidoJapan; ^3^ Research Center of HealthPhysical Fitness and SportsNagoya UniversityNagoyaJapan; ^4^ Institute of Personal Health CareKao co ltd.TokyoJapan

**Keywords:** Brachial‐ankle pulse wave velocity, Doppler ultrasound, flow‐mediated dilation

## Abstract

Endothelial dysfunction is associated with increased cardiovascular mortality and morbidity; however, this dysfunction may be ameliorated by several therapies. For example, it has been reported that heat‐induced increases in blood flow and shear stress enhance endothelium‐mediated vasodilator function. Under these backgrounds, we expect that carbon dioxide (CO_2_)‐rich water‐induced increase in skin blood flow improves endothelium‐mediated vasodilation with less heat stress. To test our hypothesis, we measured flow‐mediated dilation (FMD) before and after acute immersion of the lower legs and feet in mild warm (38°C) normal or CO_2_‐rich tap water (1000 ppm) for 20 min in 12 subjects. Acute immersion of the lower legs and feet in mild warm CO_2_‐rich water increased FMD (*P* < 0.01) despite the lack of change in this parameter upon mild warm normal water immersion. In addition, FMD was positively correlated with change in skin blood flow regardless of conditions (*P* < 0.01), indicating that an increase in skin blood flow improves endothelial‐mediated vasodilator function. Importantly, the temperature of normal tap water must reach approximately 43°C to achieve the same skin blood flow level as that obtained during mild warm CO_2_‐rich water immersion (38°C). These findings suggest that CO_2_‐rich water‐induced large increases in skin blood flow may improve endothelial‐mediated vasodilator function while causing less heat stress.

## Introduction

Several previous studies reported that various atherosclerotic risk factors, including aging, hypercholesterolemia, hyperglycemia, hyperlipidemia, hypertension, diabetes mellitus, cigarette smoking, and postmenopausal status, impaired brachial artery flow‐mediated dilation (FMD) as an index of endothelium‐mediated vasodilator function (Panza et al. 1990; Chowienczyk et al. 1992; Celermajer et al. 1993, 1994; Lieberman et al. 1994; Stroes et al. 1995; Steinberg et al. 1997; Giugliano et al. 1998; Williams et al. 1998). In addition, the endothelium dysfunction is associated with increased cardiovascular mortality and morbidity (Rossi et al. 2008; Shechter et al. 2009; Yeboah et al. 2009). However, it has been reported that endothelial dysfunction may be ameliorated by several therapies. For example, endothelial function in patients with chronic heart failure (CHF) is improved by physical training (Hornig et al. 1996) or the administration of dobutamine (Patel et al. 1999), vitamin C (Hornig et al. 1998b), angiotensin‐converting enzyme inhibitors (Hornig et al. 1998a), or l‐arginine (Hirooka et al. 1994; Rector et al. 1996). Moreover, restoration of endothelial function improves cardiac function in patients with CHF (Kihara et al. 2002), indicating that an improvement in endothelial function decreases cardiovascular risk (Modena et al. 2002; Kitta et al. 2009).

Interestingly, heat‐induced increases in blood flow and shear stress enhance endothelium‐mediated vasodilator function (Naylor et al. 2011). Accordingly, repeated systemic thermal therapy, also termed sauna therapy (60°C, 15 min for 2 weeks), improves cardiac function and clinical symptoms with an improvement in endothelial function (Kihara et al. 2002). In addition, acute leg heating (45°C, 20 min) improves impaired endothelial function and oxidative stress in patients with CHF (Inoue et al. 2012). Therefore, these thermal therapies (heating methods) may be beneficial therapeutic options for patients with hypertension, CHF, and coronary artery disease (Imamura et al. 2001; Kihara et al. 2002, 2009; Miyata et al. 2008). However, the uses of these thermal therapies are limited because a specific facility, sauna, or far‐infrared radiation is needed to perform these therapies. More importantly, heating stimulation modifies sympathetic and cardiovascular systems and in some cases increases the risk in patients with cardiovascular disease (Frishman et al. 2005; Eren et al. 2009).

Carbonated spring water containing more than 1000 ppm of carbon dioxide (CO_2_) is used for balneotherapy in patients with hypertension or peripheral occlusive arterial disease because it exerts thermal effects (CO_2_ in tap water is around 2 ppm). As a device for obtaining carbonated spring water with a CO_2_ concentration of 1000 ppm, featuring a special membrane has been developed (Kamo et al. 1985); carbonated spring water leg bathing is widely available, even for patients with cardiovascular diseases. However, the effect of carbonated water on cardiovascular system remains unknown. Interestingly, immersion of the whole body in CO_2_ rich warm water increases skin blood flow (SkBF) despite a decline in core temperature (Ito et al. 1989; Hartmann et al. 1997; Nishimura et al. 2002). On the other hand, Green et al. (Green et al. 2010) suggest that increased cutaneous blood flow is a key physiological stimulus for enhancing microvascular vasodilator function. Cutaneous thermoregulatory vasodilation induces an increase in shear stress in conduit artery and enhances conduit artery vasodilator function (Naylor et al. 2011, 2014). Under these backgrounds, we expect that mild warm CO_2_‐rich water‐induced increase in SkBF will improve endothelial‐mediated vasodilator function in conduit artery with less heat stress. Adding CO_2_ to warm normal tap water immersion will reduce the warm water temperature required to increase endothelial‐mediated vasodilator function. To test our hypothesis, we measured FMD and SkBF before and after acute immersion of both lower legs and feet in mild warm normal or CO_2_‐rich tap water (38°C).

## Materials and Methods

### Ethical approval

Written informed consent was obtained from all participants, and all study procedures were approved by the Ethics Committee on Human Research, Sapporo Medical University. The studies conformed to the Declaration of Helsinki.

### Subject's characteristics

Twelve male subjects (age, 23.8 ± 0.9 years; height, 170.8 ± 2.3 cm; weight, 63.7 ± 2.6 kg) participated in this study. The subjects were healthy, and a preparticipation questionnaire confirmed the absence of known cardiovascular disease or risk factors, with subjects receiving any medication excluded. Subjects were asked to abstain from caffeinated beverages and avoid strenuous exercise for 24 h before the experiment. The experiment was performed at least 3 h after a light meal.

### Experimental procedure

#### Immersion of the lower legs and feet in CO_2_‐rich and normal warm water

**Figure 1:**
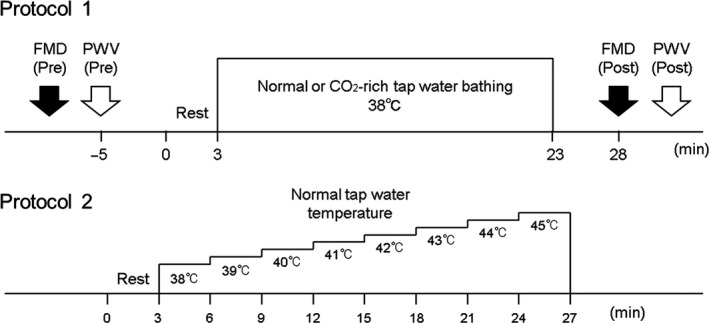
Schematic diagram of experimental protocol. Protocol 1, Immersion of lower legs and feet in mild warm CO_2_‐rich and normal tap water; Protocol 2, Heating test.

**Figure 2:**
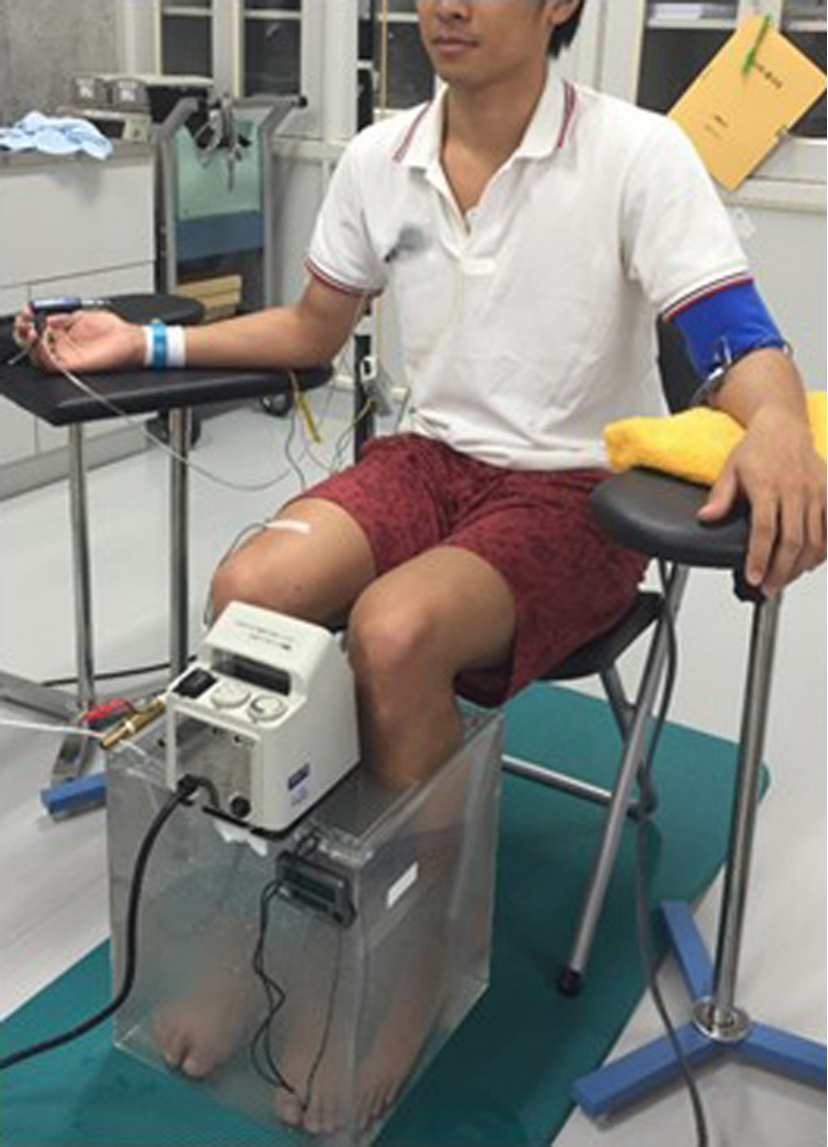
Setup of the lower leg bathing and heating test.

All experiments were performed in an air‐conditioned room maintained at a constant temperature (23–24°C). The experimental protocol and setup are shown in Figures 1 and 2. After 15 min of rest in the supine position, popliteal FMD and brachial‐ankle pulse wave velocity (baPWV) were measured. Each subject then moved to the chair and immersed both lower legs and feet in mild warm CO_2_‐rich water (38°C, 1000 ppm)(Kamo et al. 1985) or normal tap water (38°C) for 20 min in the sitting position. The CO_2_‐rich tap water was prepared by dissolving CO_2_ in tap water using tablet consisting of carbonate and fumaric acid (Kao Co., Ltd., Japan). The CO_2_‐rich tap water was colorless and odorless. In this study, we considered about the balneotherapy using mild warm CO_2_‐rich water. Thus, we used 38°C which is more than body temperature without heat stress as the experimental temperature of mild warm CO_2_‐rich or normal tap water. During bathing, the water temperature was maintained using a water thermoregulator (Thermax TM‐1, AS ONE Co., Ltd., Japan). After 20 min of bathing, subjects immediately moved to the bed, and FMD and baPWV measurements were repeated at 5 min after the bathing. The mild warm CO_2_‐rich and normal water tests were performed at the same time of the day on separate days (at least 2 days apart), and the two tests were performed in random and counterbalance order. We continuously measured arterial blood pressure (BP), cardiac output (CO), electrocardiographic variables, SkBF, and skin temperature (T_sk_). Although core body temperature is important to identify the physiological mechanism, we did not measure it to reduce any stress for the subjects. Subjective heat stress or surface temperature (partial stress) is more important for testing our hypothesis of this study. Therefore, each subject was asked to rate his current level of subjective hotness to the legs using a visual analog scale (VAS) after 20 min of bathing. The VAS ranged from 0 (not hot) to 10 (extremely hot).

#### Heating test

After the normal warm water test, we performed the heating test in 10 subjects on the same day after an interval of at least 20 min. Before the heating test, we confirmed that T_sk_ and SkBF had returned to the baseline level. In the test, the subjects rested for 3 min and then immersed their lower legs and feet in mild warm normal tap water (38°C) in the sitting position. The temperature of the normal tap water was increased by 1°C every 3 min until the subject requested no further increase or when the temperature reached 45°C, with all subjects (*n* = 10) tolerating the maximum temperature (Fig. 1). During the heating test, we continuously measured BP, CO, electrocardiographic variables, SkBF, and T_sk_. The subjects were asked to rate their current level of subjective hotness to the legs using a VAS every 3 min.

### Measurements

#### FMD and baPWV

FMD was measured according to a standard procedure (Corretti et al. 2002; Thijssen et al. 2011). Briefly, we measured the left popliteal artery FMD using a high‐resolution ultrasound machine (Vivid‐i, GE Yokogawa Medical Systems, Japan) with an 8.8‐MHz multi‐frequency linear probe. Each subject laid face down with their legs extended and a small pillow under their left ankle. The popliteal artery was measured slightly above the popliteal fossa, and an automatic cuff (5 × 84 cm^2^) was placed approximately 5 cm distal to the popliteal fossa (E‐20 rapid cuff inflator; D. E. Hokanson, USA). The location of the probe was marked on the leg skin to ensure that the site of measurement did not change between before and after bathing. The ultrasound parameters were set to optimize longitudinal B‐mode images of the lumen‐arterial wall interface. Pulsed Doppler signals were recorded at an angle of insonation of 60 degrees with a sample volume encompassing the entire width of the artery. After baseline assessment for 2 min, the cuff was inflated to 250 mmHg for 5 min and then deflated. The vessel diameter and blood flow velocity were monitored beginning 1 min prior to cuff deflation and continuing for 3 min. Images of the artery and associated velocity waveform from the Doppler ultrasound machine were stored in a computer (Pavilion H8‐1360jp/CT, HP, Japan) at a frequency of 40 Hz using a capture box (Epiphan capture tool VGA2USB LR, ARGO, Japan). The arterial diameter was analyzed using custom‐designed edge detection and wall‐tracking software (ver. 2.0.1 No. S‐13037, Takei Kiki Kogyo, Japan). The previous study (Harris et al. 2010) reported the reliability and validity of the FMD edge‐detection analysis and recommended to use software in offline analysis for the measurement of baseline and post cuff release diameters. The Doppler waveform detected by the ultrasound auto‐program was automatically traced using the same software utilized to calculate the mean blood velocity. The synchronized diameter and velocity were analyzed at 40 Hz. The peak diameter after cuff deflation (Dmax) was automatically detected according to an algorithm that identified the maximum bracket of data subsequent to performance of a moving window smoothing function, as reported previously (Black et al. 2008). FMD was calculated as the percentage increase in Dmax from the baseline diameter (Dbase) using the formula FMD = ([Dmax−Dbase]/Dbase) × 100 (%). Shear rate (SR) was calculated as four times the velocity divided by the diameter, and the SR area under the curve (SR_AUC_) was calculated as the area from the time of deflation to the time of Dmax (peak time). Normalization of FMD to SR (Normalized‐FMD) was calculated by dividing FMD by SR_AUC_ (Atkinson et al. 2009).

After the measurement of FMD, the subject changed from a prone to a supine position, and baPWV as an index of systemic arterial stiffness was measured using a noninvasive vascular profiling system (Form PWV/ABI; Omron Healthcare, Japan) according to a standard procedure (Sugawara et al. 2014). Bilateral brachial and ankle arterial pressure waveforms were stored for 10 sec using extremity cuffs connected to an oscillometric pressure sensor wrapped around both arms and ankles. The baPWV was calculated from the distance between two arterial recording sites divided by the transit time. The distance travelled by the pulse waves was assessed by a random zero‐length measurement over the surface of the body using a nonelastic tape measure. Pulse wave transit time was determined from the time delay between the proximal and distal “foot” waveforms. The foot of the wave was identified as the commencement of the sharp systolic upstroke, which was detected automatically. In this study, the coefficient of variation for intra‐observer reproducibility of baPWV was <5%.

### Vascular variables, SkBF, and T_sk_

An electrocardiogram was obtained using a three‐lead electrocardiograph (FA‐DL‐150, 4assist, Japan), and heart rate (HR) was calculated from each R‐R interval. BP and CO were acquired using finger photoplethysmography from the middle finger of the right hand (Finometer, Finapres Medical Systems BV). Arterial systolic and diastolic BP (SBP and DBP, respectively) were determined from the BP waveform signal. SkBF and T_sk_ were measured on the immersed lower leg by the laser‐Doppler flowmetry system (MoorVMS‐LDF, Moor Instruments, UK). The laser‐Doppler probe (VP7a/T, Moor instruments, USA) including a laser Doppler and thermo‐sensor was attached at a distance of 1 mm from the skin surface, because the skin surface to be measured must be touched with CO_2_‐rich water for it to be influenced by CO_2_ (Nishimura et al. 2002). All signals were sampled at a frequency of 1 kHz through an analog‐to‐digital converter (Power Lab 16/s, ADInstruments, Australia) and stored in a computer (QOSMIO‐F40, Toshiba, Japan). The parameters were averaged over 3 min at rest and over the last minute of warm CO_2_‐rich or normal water bathing. Cutaneous vascular conductance (CVC) was calculated as the ratio of SkBF to brachial mean blood pressure. During the heating test, the data were averaged 1 min at rest and over the last 30 sec of immersion at each temperature.

### Statistics

To determine the number of subjects needed for the study, we performed a power test using FMD data in CO_2_‐rich water immersion between pre‐ and postconditions in three subjects in the prestudy. In the power‐paired t‐test (*δ *= 2, *σ *= 2, *α* level = 0.05, power = 0.8) calculation, sample size should be *n* > 10 to obtain a significant difference. All values are presented as the mean ± SE. Two‐way analysis of variance (ANOVA) with repeated measures was performed. The condition (CO_2_‐rich or normal water) was one factor considered in the analysis, with time used as the second factor. To compare parameters between before and after bathing, a paired *t*‐test was used. Dunnett's test was used to evaluate differences between the baseline data and those of during bathing when a significant interaction or main effect was identified in ANOVA. Comparisons of parameters between the two conditions were achieved using a paired *t*‐test. The FMD, which was controlled changes in baseline diameter (Corrected FMD), was also calculated using an analysis of covariance (ANCOVA), as previously utilized (Atkinson and Batterham 2013). Differences between means were evaluated using a post hoc LSD test. The relationship between FMD and SkBF was assessed by Spearman's correlation coefficients. The SPSS (11.5; SPSS, Tokyo, Japan) statistical software package was used to perform the statistical analyses. Statistical significance was set at *P* < 0.05.

## Results

Acute immersion of the lower legs and feet in both normal and CO_2_‐rich warm water increased T_sk_ to approximately 38°C, whereas these conditions slightly increased HR, CO, SBP, and DBP without statistical significance (Table 1). Both immersion protocols also gradually increased SkBF and CVC; however, the change in SkBF and CVC were much larger in mild warm CO_2_‐rich water than that in normal tap water (*P* < 0.01). Despite the large increase in SkBF during immersion in CO_2_‐rich water, the value of VAS was still low (1.3 ± 0.4), that is, not significantly different from that in mild warm normal tap water. In the heat test, during lower leg bathing, the temperature of warm normal tap water was required to reach approximately 43°C to be able to achieve the same SkBF level as that obtained during immersion in mild warm normal tap water (38°C) (Table 2). Then HR and VAS increased significantly from 38°C mild warm water (HR, 56 ± 2–64 ± 3 bpm; VAS, 1.1 ± 0.4–5.2 ± 0.6), while BP was unchanged.

**Table 1:** Vascular variables, skin temperature and skin blood flow, and VAS during lower leg bathing

	Pre	Bathing at 20 min	Two‐way ANOVA		
			Time	Condition	Time × Condition
HR (bpm)					
Normal	58.1 ± 1.9	58.5 ± 1.9	0.09	0.65	0.08
CO_2_	56.1 ± 2.2	58.7 ± 1.8			
CO (L/min)					
Normal	4.8 ± 0.3	5.0 ± 0.4	0.50	0.15	0.29
CO_2_	4.4 ± 0.1	4.7 ± 0.2			
SBP (mmHg)					
Normal	118.4 ± 2.9	119.8 ± 3.2	0.11	0.12	0.21
CO_2_	121.3 ± 2.2	125.2 ± 3.2			
DBP (mmHg)					
Normal	67.0 ± 2.2	67.3 ± 2.5	0.13	0.19	0.28
CO_2_	69.0 ± 1.6	70.8 ± 1.7			
Tsk (°C)					
Normal	27.6 ± 0.7	37.8 ± 0.7^b^	**<0.01**	0.30	0.44
CO_2_	27.6 ± 0.5	38.2 ± 0.7^b^			
SkBF (%)					
Normal	100.0 ± 0.0	254.3 ± 35.0^b^	**<0.01**	**<0.01**	**<0.01**
CO_2_	100.0 ± 0.0	643.5 ± 52.6^a^, ^b^			
CVC (%)					
Normal	100.0 ± 0.0	252.4 ± 35.3^b^	**<0.01**	**<0.01**	**<0.01**
CO_2_	100.0 ± 0.0	618.3 ± 49.8^a^, ^b^			
VAS					
Normal	N/A	0.8 ± 0.3	N/A	N/A	N/A
CO_2_	N/A	1.3 ± 0.4	N/A	N/A	N/A

Values are means ± SE; *n* = 12 subjects.

HR, heart rate; CO, cardiac output; SBP, systolic blood pressure; DBP, diastolic blood pressure; Tsk, skin temperature; SkBF, skin blood flow; VAS, visual analog scale; Normal, Normal tap water; CO_2_, CO_2_‐rich tap water;

a Post hoc significantly different between Normal and CO_2_ at *P* < 0.05.

b Post hoc significantly different from Pre at *P* < 0.05.

**Table 2:** Vascular variables, skin temperature and skin blood flow, and VAS during heating test

	Rest	Normal tap water bathing								One‐Way ANOVA
		38°C	39°C	40°C	41°C	42°C	43°C	44°C	45°C	Time
HR (bpm)	58.3 ± 2.3	56.4 ± 2.4	59.2 ± 2.2	58.9 ± 2.3	60.2 ± 2.0	58.4 ± 1.9	64.2 ± 3.1^a^	67.0 ± 4.2^a^	66.6 ± 1.9^a^	**<0.01**
SBP (mmHg)	125.5 ± 2.6	125.2 ± 3.4	124.3 ± 3.4	124.6 ± 3.2	128.3 ± 3.8	127.5 ± 3.7	128.9 ± 3.2	131.5 ± 3.7^a^	132.0 ± 4.4^a^	**<0.01**
DBP (mmHg)	73.0 ± 1.6	70.8 ± 1.9	69.9 ± 1.9	70.4 ± 2.3	72.7 ± 2.2	72.5 ± 2.5	73.1 ± 2.2	74.5 ± 2.4	74.6 ± 2.6	**<0.01**
Tsk (°C)	27.5 ± 1.0	37.5 ± 0.1^a^	38.2 ± 0.1^a^	39.2 ± 0.2^a^	39.8 ± 0.2^a^	40.6 ± 0.3^a^	41.4 ± 0.3^a^	42.3 ± 0.3^a^	43.1 ± 0.3^a^	**<0.01**
%SkBF (%)	15.7 ± 3.2	34.4 ± 6.6	41.4 ± 7.0	42.1 ± 5.0	48.0 ± 7.2	73.2 ± 7.8	135.4 ± 21.7^a^	255.3 ± 41.2^a^	316.9 ± 48.7^a^	**<0.01**
VAS	N/A	1.1 ± 0.4	1.5 ± 0.4^b^	2.5 ± 0.4^b^	3.5 ± 0.5^b^	4.1 ± 0.5^b^	5.2 ± 0.6^b^	6.5 ± 0.5^b^	7.2 ± 0.5^b^	**<0.01**

Values are means ± SE; *n* = 10 subjects.

HR, heart rate; SBP, systolic blood pressure; DBP, diastolic blood pressure; Tsk, skin temperature; SkBF, skin blood flow; %SkBF, SkBF/SkBF at 20 min during CO_2_‐rich water×100; VAS, visual analog scale.

a Post hoc significantly different from Rest at *P* < 0.05.

b Post hoc significantly different from 38°C at *P* < 0.05.

**Figure 3:**
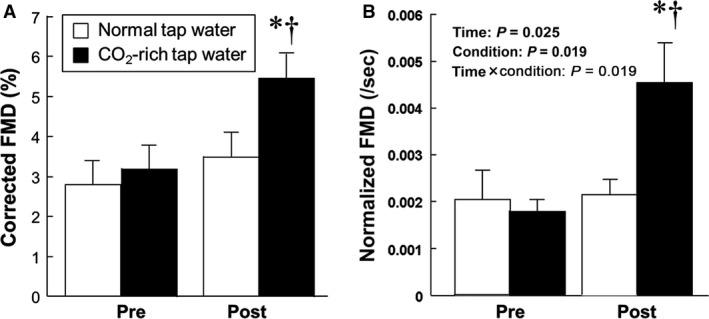
Corrected FMD (A) and normalized‐FMD (B) before and after lower leg bathing. *P < 0.05 versus pre at mild warm CO_2_‐rich water, ^†^ *P* < 0.05 versus post at normal warm tap water. FMD, flow‐mediated dilation.

**Figure 4:**
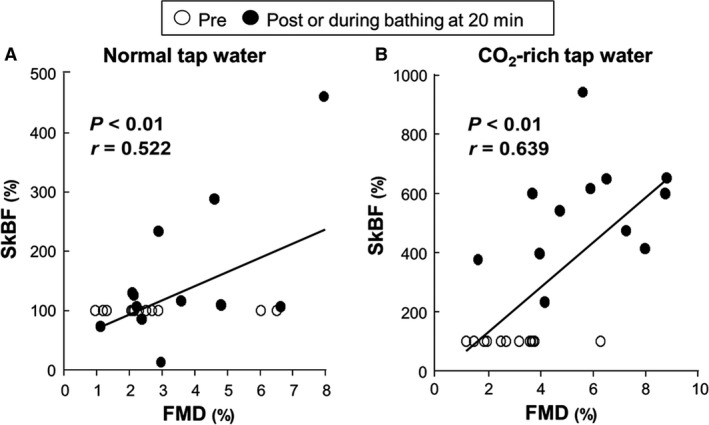
Correlation between relative changes in FMD and SkBF during the immersion of normal (A) or CO_2_‐rich tap water (B). FMD, flow‐mediated dilation; SkBF, skin blood flow. FMD was measured at pre‐ (○) and postbathing (●), and SkBF was measured at pre (○), and during bathing at 20 min as the postdata (●).

During acute lower leg hyperemia, the baseline diameter of the popliteal artery and its peak time to maximal vasodilation were unchanged by acute immersion of the lower leg and foot in either warm normal tap or CO_2_‐rich water (Table 3). In addition, there was no significant difference in the change in SR_AUC_ between the conditions. Nevertheless, acute immersion in mild warm CO_2_‐rich water increased FMD and corrected FMD (Table 3 and Fig. 3A, *P* < 0.01) despite the lack of change in FMD in warm normal tap water. In addition, the difference in FMD between the two conditions was enhanced by normalization of SR_AUC_ (Fig. 3B, *P* < 0.01). Similarly, baPWV significantly decreased after immersion in warm CO_2_‐rich water (Table 3). Importantly, FMD was significantly associated with change in SkBF regardless of conditions (Fig. 4, *P* < 0.01).

**Table 3:** Peak time, Dbase, Dmax, and SR_AUC_ during FMD and PWV before and after bathing

	Pre	Post	Two‐way ANOVA		
			Time	Condition	Time × Condition
Peak time (sec)					
Normal	44.2 ± 4.1	49.0 ± 5.8	0.76	0.41	0.26
CO_2_	46.9 ± 4.2	39.4 ± 4.0			
Dbase (mm)					
Normal	5.96 ± 0.15	5.92 ± 0.15	0.20	0.26	0.57
CO_2_	5.89 ± 0.17	5.87 ± 0.17			
Dmax (mm)					
Normal	6.11 ± 0.14	6.13 ± 0.16	**<0.05**	0.85	**<0.01**
CO_2_	6.06 ± 0.17	6.20 ± 0.17^b^			
srauc (10^3^/sec)					
Normal	16.4 ± 1.9	17.0 ± 1.7	0.17	0.58	0.13
CO_2_	17.8 ± 1.4	14.0 ± 1.2			
FMD (%)					
Normal	2.7 ± 0.1	3.6 ± 0.6	**<0.01**	**<0.01**	**<0.05**
CO_2_	3.0 ± 0.4	5.7 ± 0.7^a^, ^b^			
PWV (cm/sec)					
Normal	1108 ± 28	1094 ± 37^b^	**<0.01**	0.11	0.22
CO_2_	1125 ± 27	1066 ± 25^b^			

Values are means ± SE; *n* = 12 subjects. Peak time, time to peak vasodilation after deflation; Dbase, baseline diameter; Dmax, maximum diameter; SR_AUC_, shear rate area under the curve; FMD, flow‐mediated dilation; PWV, pulse wave velocity; Normal, Normal tap water; CO_2_, CO_2_‐rich tap water.

a Post hoc significantly different between Normal and CO_2_ at *P* < 0.05.

b Post hoc significantly different from Pre at *P* < 0.05.

## Discussion

Acute immersion of the lower legs and feet in mild warm normal tap water did not alter FMD. By contrast, acute immersion in warm CO_2_‐rich water at the same temperature increased FMD. These findings indicate that immersion in mild warm CO_2_‐rich water improves endothelium‐mediated vasodilation with less heat stress. Thus, mild warm CO_2_‐rich water may be a useful therapeutic option for improving endothelial function especially for patients with physiological sensitivity to heat or with cardiovascular disease which is deteriorated by heat stress.

Some previous studies demonstrated that repeated (Imamura et al. 2001; Kihara et al. 2002, 2009) and acute thermal therapy (Inoue et al. 2012) improved impaired endothelial function in patients with CHF. Similarly, in this study, acute immersion of the lower leg and foot in mild warm CO_2_‐rich warm water improved both endothelial function (FMD) and arterial stiffness (baPWV) (Table 3 and Fig. 3). Importantly, immersion in mild warm CO_2_‐rich water may not cause heat stress because the VAS during CO_2_‐rich water immersion is still low (1.3 ± 0.4) and this value is not significantly different from that during mild warm normal tap water immersion (0.8 ± 0.3). In addition, previous study (Nishimura et al. 2002) reported that CO_2_‐rich water does not increase core body temperature despite an increase in SkBF. Unfortunately, core body temperature was not measured in this study. However, the VAS data should be associated with core body temperature or subjective heat stress. Thus, these findings suggest that adding CO_2_ to warm normal tap water immersion reduces heat stress required to increase endothelium‐mediated vasodilator function.

It has been considered that thermal therapy‐induced improvement in endothelial function may be associated with the relationship between central thermal drive and peripheral adaptation during heat stimulation (Green et al. 2004) via endothelial nitric oxide synthase (eNOS) upregulation (Kihara et al. 2002) or decreases in oxidative stress contribute to the increase in NO bioavailability and vascular endothelial growth factor upregulation (Inoue et al. 2012). Therefore, improvement in endothelial‐mediated vasodilator function after warm CO_2_‐rich water bathing may be associated with changes in NO production or oxidative stress. In this study, unfortunately, we did not measure any NO or oxidative stress markers. We need to perform further investigations to examine the effect of acute immersion in mild warm CO_2_‐rich water on NO production or oxidative stress.

However, thermal therapy or heat stimulation‐induced central thermal drive and peripheral adaptation does not occur similarly during mild warm CO_2_‐rich water immersion. One possible mechanism for improvement in endothelial vasodilator function by immersion in CO_2_‐rich water may be associated with increase in SkBF. In this study, indeed, FMD was significantly associated with SkBF regardless of conditions (Fig. 4). Indeed, the previous study (Green et al. 2010) suggests that increased cutaneous blood flow is a key physiological stimulus for enhancing microvascular vasodilator function. Cutaneous thermoregulatory vasodilation induces an increase in shear stress in conduit artery and enhances conduit artery vasodilator function (Naylor et al. 2011, 2014). In addition, endothelium‐derived hyperpolarizing factor (EDHF)‐mediated activation of calcium‐activated potassium (KCa) channels plays a major role in cutaneous thermal hyperemia (Brunt and Minson 2012). Activation of KCa channels also affects NO signaling (Bolotina et al. 1994; Wellman et al. 1996). These findings suggest that increase in cutaneous blood flow improves endothelium‐mediated vasodilation. More interestingly, in this study, arterial stiffness (baPWV) was improved acutely via carbonated water‐induced skin vasodilation. This result is supported by the previous study (Bellien et al. 2010) demonstrating that arterial stiffness is also regulated by the endothelium through the release of both NO and cytochrome‐related EDHF. Our results are therefore the first, to the best of our knowledge, to indicate that acute increases in the microvascular bed may contribute to the improvement in endothelium‐mediated vasodilator function via immersion in mild warm CO_2_‐rich water, and this phenomenon occurs independently of heat stress. However, we need further investigations to identify its physiological mechanism.

It has been reported that immersion of the lower legs and feet in CO_2_‐water increases cutaneous blood flow (Ito et al. 1989; Hartmann et al. 1997). During lower leg bathing, the subjects may inhale CO_2_ gas that had been released from the bath water (Yorozu et al. 1985). However, the CO_2_ concentration of the air inhaled from bath water containing 1000 ppm CO_2_ is not sufficient to cause hypercapnia (Nishimura et al. 2002). Furthermore, the denervated skin exhibits vasodilative effects (Ito et al. 1989). In addition, an elevation of subcutaneous CO_2_ tension occurs only in the skin immersed in CO_2_‐rich water (Komoto et al. 1986). CO_2_‐induced vasodilation is associated with extracellular acidosis which might reduce the contractility of the vascular smooth muscle, leading to vasodilation (Vanhoutte and Clement 1968). Taken together, CO_2_ may affect skin vessels directly from the water, and CO_2_‐induced extracellular acidosis causes vasodilation in skin vessels. Although an alteration in lower leg muscle blood flow may affect FMD, the effect of warm CO_2_‐rich water on muscle blood flow remains unknown.

There are several limitations to this study. Similar to our results, a previous study reported that CO_2_‐rich water bathing increases cutaneous blood flow and decreases core body temperature while elevating thermal sensation (Nishimura et al. 2002). However, we did not measure core temperature to reduce any stress for the subject as possible. Thus, the heat stress associated with this therapy remains unclear. However, the VAS for level of subjective hotness during CO_2_‐rich warm water bating was low (approximately 1.3, compared to approximately 0.8 for warm normal tap water bathing), whereas a warm normal tap water temperature must reach approximately 43°C to increase the same SkBF level as that obtained during 38°C warm CO_2_‐rich water bathing. Secondly, FMD and PWV were measured during supine position immediately after the immersion at seated position. Thus, the acute position change may influence both, measurements as well as cardiovascular system. However, there are no differences in the experimental procedure between two protocols (CO_2_‐rich water vs. normal tap water). Thus, this limitation could not change the relationship in both PWV and FMD between different conditions. Last considerable limitation pertains to the laser Doppler measurement. One limitation regarding this technique is that we did not measure maximal SkBF to reduce subject's stress; thus, we used the relative change in SkBF from the baseline value. Also, different values may result simply from placement of the probe and the density of underlying vessels because this technique only measures 1 mm from skin as the previous study (Nishimura et al. 2002).

### Clinical perspectives

The acute improvement in flow‐mediated dilation upon CO_2_‐rich warm water immersion was similar to the effects of thermal therapy. This therapy using CO_2_‐rich mild warm water does not require a specific facility. Thus, it is an inexpensive therapy that can be performed easily and repeatedly at any location. More importantly, immersion of the lower legs and feet in CO_2_‐rich warm water could be performed with less heat stress to improve endothelial‐mediated vasodilator function; thus, this therapy should not induce adverse effects associated with thermal therapy including heat stress‐induced worsening of clinical symptoms, skin burns, dehydration, and arrhythmias. Thus, mild warm CO_2_‐rich water may be a useful therapeutic tool for patients with hypertension or peripheral occlusive arterial disease while causing less heat stress.

In conclusion, immersion of the lower legs and feet in mild warm CO_2_‐rich water improves endothelial‐mediated vasodilator function and arterial stiffness while causing less heat stress. This improvement of peripheral vasculature may be due to mild warm CO_2_‐rich water‐induced large increase in SkBF. Thus, mild warm CO_2_‐rich water may be a useful therapeutic tool for patients with hypertension or peripheral occlusive arterial disease while causing less heat stress.

## Conflict of Interest

Shohei Kimura, Yasuhiro Shidahara, Tomomi Ishii, and Michinari Kudoh are employees of Kao co ltd. The other authors report no conflicts.
